# Treatment of giardiasis in dogs: field clinical study to confirm the efficacy, safety, and acceptance of a metronidazole-based flavored oral suspension

**DOI:** 10.1186/s13071-025-06797-w

**Published:** 2025-05-12

**Authors:** Sloane Jones, Philippe Briantais, Cristiano Von Simson, Eleonore De Meyrignac, Laure Poincelot, Delphine Rigaut

**Affiliations:** 1 Virbac Corporation, 1301 Solana Boulevard, Westlake, TX 76262 USA; 2 Virbac, 13ème Rue, LID, 06511 Carros, France

**Keywords:** *Giardia*, Metronidazole, Dog, Canine, Treatment

## Abstract

**Background:**

*Giardia duodenalis* is a prevalent gastrointestinal parasite in dogs, causing diarrhea, vomiting, and weight loss. Metronidazole is a common treatment for this infection. This study aimed to evaluate the efficacy, safety, and acceptance of a flavored liquid metronidazole oral suspension in treating *G. duodenalis* in naturally infected dogs.

**Methods:**

A double-masked, vehicle-controlled, randomized, multi-center clinical field trial was conducted. Client-owned dogs with confirmed *G. duodenalis* infections were enrolled and randomized into AYRADIA-treated and control groups. The AYRADIA group received the metronidazole suspension at 0.2 ml/kg twice daily for 5 days, while the control group received a flavored vehicle suspension without metronidazole. Fecal samples were collected before and after treatment to assess *G. duodenalis* cyst counts. Clinical examinations and owner assessments were also performed to evaluate safety and treatment acceptance.

**Results:**

The study enrolled 180 dogs, with 129 included in the efficacy analysis. AYRADIA treatment resulted in a 99.92% reduction in *G. duodenalis* cyst counts, significantly higher than the reduction in the control group. Adverse events were similar between both groups (10%), mainly consisting of diarrhea and vomiting. The treatment was readily accepted by 99% of dogs.

**Conclusions:**

AYRADIA, administered at 0.2 ml/kg twice daily for 5 days, is highly effective in treating *G. duodenalis* infections in dogs. The treatment demonstrated a positive safety profile and excellent acceptance. This flavored oral suspension offers a valuable and convenient option for veterinarians managing giardiasis in dogs.

**Graphical Abstract:**

**Figure:**
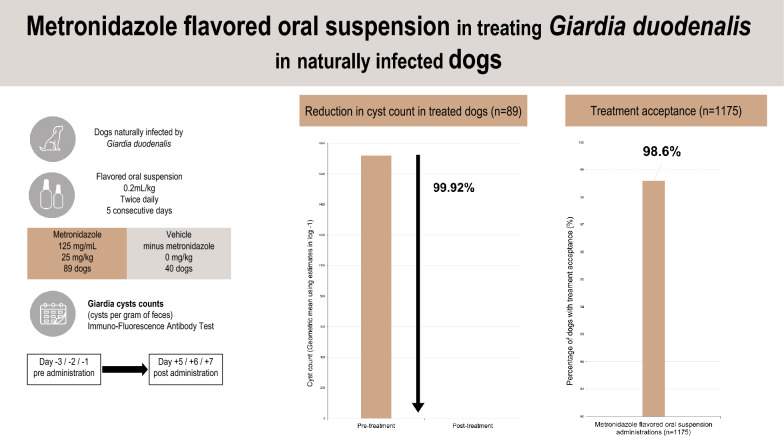

## Background

*Giardia duodenalis* is a commonly found gastrointestinal protozoan parasite in dogs. Clinical signs of *G. duodenalis* infection include acute and chronic diarrhea, vomiting, weight loss, and apathy [1–3]. Infection with *G. duodenalis* poses both health and zoonotic risks, especially in environments like kennels, shelters, and multi-pet households. The treatment of this disease is crucial due to the potential zoonotic risk and to the economic and sanitary impact in breeding establishments and shelters [4–6]. Treatment success can be a challenge due to the life cycle of *G. duodenalis* and relies on drug administration in combination with strict hygiene measures [7, 8]. Metronidazole is a nitroimidazole with antiprotozoal and antibacterial activity [8, 9]. Due to its efficacy, metronidazole is widely used as a therapeutic option [10, 11].

Virbac launched the first European Medicines Agency (EMA)- and Food and Drug Administration (FDA)-approved flavored liquid metronidazole formulation for veterinary use. The product is a flavored liquid metronidazole oral suspension for dogs. The oral suspension contains metronidazole at 125 mg/ml. The product's commercial name, ERADIA or AYRADIA, varies by region. This clinical field study aimed to assess the efficacy, safety, and acceptance of AYRADIA, intended to treat *G. duodenalis* infection in dogs under field conditions.

## Methods

The study was a double-masked, vehicle-controlled, randomized, and blocked, multi-center clinical field trial evaluating the efficacy and safety of AYRADIA (Virbac) for use in dogs naturally infected with *G. duodenalis*.

### Animals

Client-owned dogs, naturally infected with *G. duodenalis,* were enrolled in the study. The study was conducted in 10 veterinary centers in three European countries (Germany, Hungary, and Portugal). The housing and climatic conditions were similar in the selected sites in Europe (Northern hemisphere), and therefore representative of the conditions in the United States. Similarly, the parasites identified, all susceptible to metronidazole treatment, were comparable between the European sites and the United States [10].

The dogs were privately owned and/or maintained in intensely managed facilities (shelters or non-breed-specific rescue groups). For facility animals, an owner could have a maximum of six dogs enrolled and followed during the same time period. Enrolled dogs were generally in good health based on a veterinary physical exam, and dogs with diarrhea were enrolled if the veterinarian deemed their condition suitable. Dogs had a positive SNAP^®^ Giardia Test (IDEXX, USA). Pre-treatment fecal samples were collected by the owners on days −3, −2, and −1 and sent to a central laboratory for immunofluorescence antibody testing (IFAT). Exclusion criteria encompassed pregnancy, lactation, concurrent clinical trial participation, and pre-existing medical conditions that could confound study outcomes. All dogs underwent a canine parvovirus rapid test prior to inclusion, with positive results leading to exclusion. Other potential co-infections were not assessed. If none of the three pre-treatment fecal samples had an IFAT result of at least 750 *G. duodenalis* cysts per gram of feces (CPG), the dog was removed from the study after its inclusion and treatment. Animal care and management practices were consistent across all study sites and adhered to recommendations for preventing animal-to-animal transmission of *G. duodenalis* [7, 8]. Recommendations included the cleaning and drying of the environment (blankets, bedding, etc.) by means of steam-cleaning or use of commercially available disinfectants, cleaning and drying of food and water containers daily with boiling water, performing proper disposal of feces (daily removal of feces and disposal of fecal material in municipal waste), and bathing the dogs using gloves and shampoo and focusing on the peri-anal region on day 0 and day 5.

### Treatment

The enrolled dogs were randomized in a ratio of 2:1 to either the AYRADIA-treated or control group. Beginning at day 0, all dogs received either AYRADIA or the control product at a dose of 0.2 ml/kg twice daily for five consecutive days. In the AYRADIA-treated group, the dogs received AYRADIA (Virbac, France), a flavored oral suspension containing 125 mg/ml of metronidazole. Each dog received 25 mg/kg of metronidazole twice daily. In the control group, the dogs received a vehicle-flavored oral suspension (equivalent to the final formulation of the product minus metronidazole). The prescribed dose was administered by the owner directly into the mouth or top-dressed onto the food.

### Monitoring and samples

Fecal samples were collected by the owner on days −3, −2, and −1 pre-treatment and on days +5, +6, and +7 post-treatment. The fecal samples were stored in the refrigerator until being returned to the veterinary clinic and shipped to the designated central laboratory.

Fecal samples were analyzed using a direct IFAT for the detection of *G. duodenalis*. The method for performing the *Giardia* cyst counts was developed from two publications (Xiao, 1993 [12] and Barbecho, 2018 [13]) and the instructions included with the three kits used (Mini Parasep^®^ SF Kit, Merifluor Cryptosporidium/Giardia^®^ Kit, Mini Parasep^®^ Faecal Parasite Concentrator Kit). The cyst count was calculated as *G. duodenalis* CPG.

A veterinary exam including hematology, biochemistry, and urine analyses was performed on day −3 (pre-administration) and day +5 (post-administration).

The dogs were subject to owner observation for health-related changes twice daily from day 0 to day 4, and once daily from day 5 to day 7.

### Statistical analysis

All statistical analyses were performed using validated SAS^®^ statistical analysis software (version 9.4; SAS Institute Inc., Cary, NC, USA). Statistical significance was declared at a 5% two-sided level (*P* = 0.05). To demonstrate effectiveness, the AYRADIA-treated group needed to show a ≥ 90% reduction in post-treatment cyst counts compared with baseline levels and to show a significant difference over its vehicle in terms of post-treatment cyst counts. The percentage reduction in cyst counts in the AYRADIA-treated group was calculated using the formula: percentage reduction = ([baseline geometric mean − post-treatment geometric mean]/baseline geometric mean) × 100. Cyst counts were calculated as the geometric mean of three determinations (mean of the natural logarithmic counts).

The secondary objective was to assess the safety and the treatment acceptance.

## Results

### Population

A total of 180 dogs were enrolled in the study and included in the safety population. The safety population comprised any dog that received at least one dose of AYRADIA or the control product. Dogs from intensely managed facilities (shelters or non-breed-specific rescue groups) did not represent more than two thirds of the study population.

The majority of dogs were mixed-breed dogs (76.1%) with a mean age of 3.1 years (0.1–15.2) and relatively equal proportion of males and females, 49.4% and 50.6%, respectively, with 36.7% neutered. The mean body weight was 13.6 kg (2.0–37.2).

A total of 129 dogs were included in the efficacy population. The most common reasons for a dog's exclusion from the effectiveness assessment included the following: none of the three pre-treatment CPG counts being at least 750 *G. duodenalis* CPG (21 dogs), deviations in the amount of product administered by the owner, concomitant disease that would have impacted the study outcome, and missing fecal samples.

In the efficacy population (*n* = 129), 89 dogs were included in the AYRADIA-treated group and 40 in the control group. In the efficacy population, although all dogs tested positive for *G. duodenalis* using both the IDEXX SNAP^®^ Giardia Test and the fecal IFAT, only 10.85% of dogs displayed diarrhea as a clinical sign of *G. duodenalis* infection (14/129).

### Percent reduction of cyst excretion

In the AYRADIA-treated group (*n* = 89), the mean baseline cyst count was 1510.0 CPG (8.9–63,358.3), which was reduced to a mean post-treatment cyst count of 1.6 CPG (0–1776).

In the control group (*n* = 40), the mean cyst count was reduced from 2389.5 CPG (12–63,358.3) before administration to 268.8 CPG (0–41,163.5) after administration.

AYRADIA was significantly more efficacious than the vehicle, reducing baseline cyst counts 220 more effectively (*P* < 0.001).

**Fig. 1:**
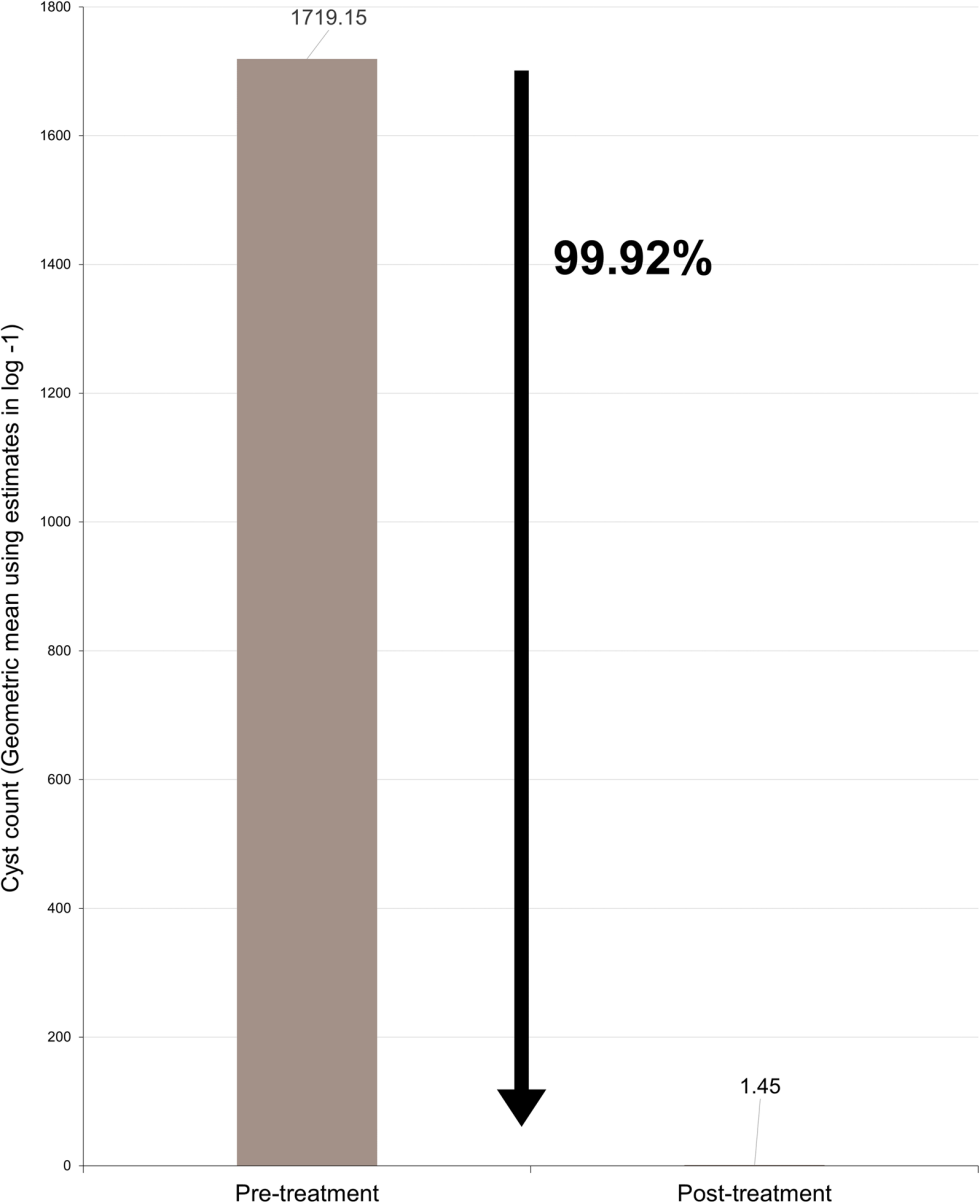
Mean cyst count in feces in AYRADIA-treated group (*n* = 89)

The percentage reduction in cyst excretion before and after treatment, calculated for the AYRADIA-treated group, showed effectiveness of 99.92% (Fig. 1).

### Improvement of clinical diarrhea

Of the dogs in the AYRADIA-treated group, 12% (11/89) had diarrhea at the baseline physical exam. Following treatment with AYRADIA, 81.8% (9/11) of these dogs no longer had diarrhea. This change from diarrhea pre-treatment to normal stool post-treatment may be due to the effects of metronidazole.

### Safety evaluation

Safety was assessed descriptively by comparing the two groups for occurrence of adverse events (AEs based on the Veterinary Dictionary for Drug Regulatory Activities [VeDDRA]).

The frequency of adverse events was similar between the two groups (10%).

Investigations, including hematology, biochemistry, and urine analyses, were performed after administration (day +5). Neutropenia or elevated liver enzymes, signs expected after metronidazole administration, were observed in 0.8% of dogs in the AYRADIA-treated group. Neurological signs were not observed. No animals died or were euthanized during the study.

Digestive tract disorders were reported in 9.2% of dogs in the AYRADIA-treated group and 6.7% in the control group. Diarrhea and emesis were the most frequently reported signs post-administration in both groups. Vomiting was reported in five dogs (4.2%) in the AYRADIA-treated group and two dogs (3.3%) in the control group.

### Administration details

With regard to administration, 91.1% of the study treatments were administered directly into the dogs’ mouths, and 8.9% of study treatments were top-dressed on the dogs’ food.

**Fig. 2:**
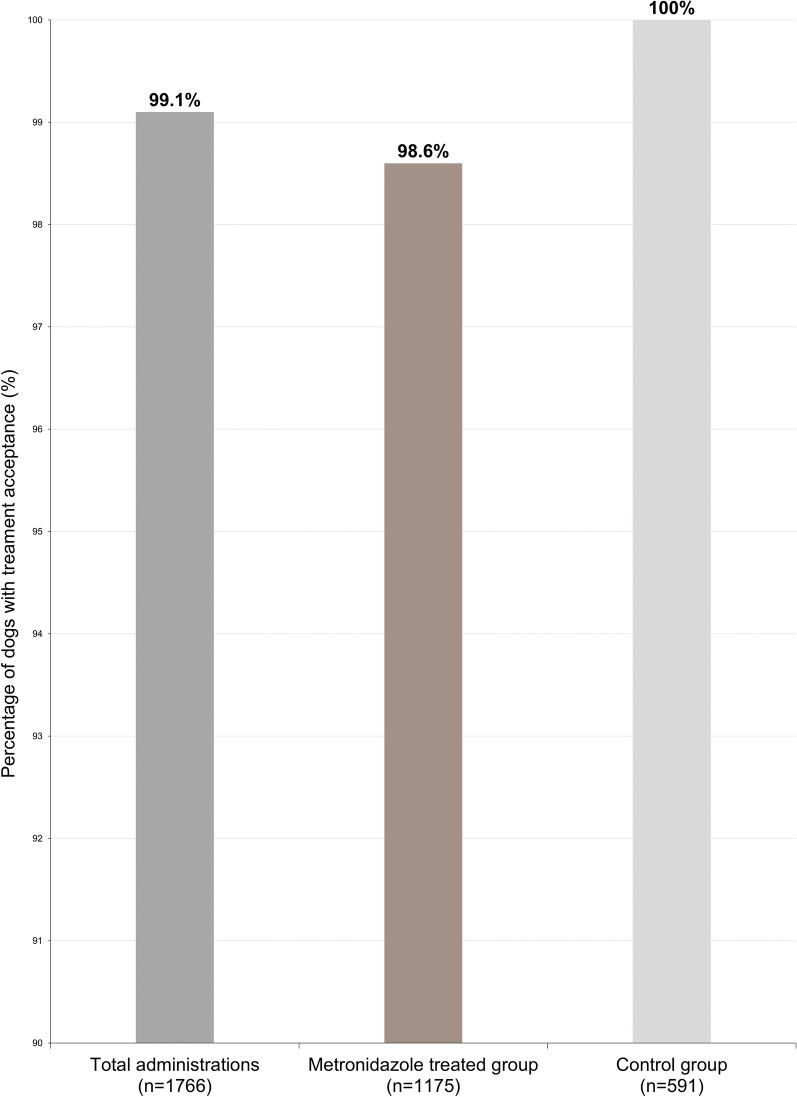
Treatment acceptance

In addition, 99.1% of the administered study treatments were readily accepted by the dogs (Fig. 2).

## Discussion

This study demonstrated 99.92% efficacy for AYRADIA administered at 0.2 ml/kg twice daily for 5 days in eliminating *G. duodenalis* in naturally infected dogs.

The treatment of *G. duodenalis* infection in dogs, the primary indication for AYRADIA, is strongly supported by the observed marked reduction in cyst shedding. This reduction serves as a direct indicator of successful treatment at the individual level. Furthermore, this substantial decrease in cyst excretion plays a critical role in mitigating environmental contamination, a key determinant in controlling *G. duodenalis* transmission, particularly within multi-dog populations and households, and has implications for public health considerations [4, 5, 7].

This study integrated metronidazole treatment with hygiene measures, as recommended by global established guidelines [7, 8, 14]. These recommended measures included cleaning the dog with a shampoo, focusing on the peri-anal region, cleaning and disinfecting floors, and cleaning and drying toys, food bowls, clothing, and pet bed. The observed decrease in mean cyst count within the control group could be attributed to the implementation of recommended hygiene measures. The critical importance of hygiene measures must be emphasized as an integral component of successful giardiasis treatment for preventing reinfection in treated dogs and minimizing the risks of relapse and transmission to other animals and human contacts [7, 8, 14].

AYRADIA demonstrated a favorable safety profile, with the incidence of mild and transient gastrointestinal adverse events (diarrhea and emesis) being comparable between the treated and control groups. This suggests that these events were likely associated with individual gastrointestinal sensitivity, rather than a direct consequence of metronidazole administration [8, 14]. This finding indicates that AYRADIA could be administered with a measured risk of adverse reactions in dogs.

Palatability is a crucial factor influencing treatment, both compliance and efficacy. In this trial, AYRADIA demonstrated excellent acceptance (99%). While most dogs readily consume AYRADIA delivered directly into the mouth, the liquid formulation offers the flexibility of administration with food, which can be particularly beneficial for reluctant dogs. The high level of acceptance observed with AYRADIA, despite the inherent taste challenge of this molecule, underscores the high quality standards met in the flavored liquid formulation and the synergistic effects of the ingredients in enhancing palatability [15].

The absence of a direct comparator group could be advanced as a potential limitation in the study design. However, the robust efficacy demonstrated by AYRADIA, coupled with the availability of extensive published literature evaluating and comparing various anti-*Giardia* agents, provides a comprehensive context for interpreting the findings of this study. A similar study published in 2018 demonstrated 92% efficacy for ERADIA [16]. In this study, metronidazole treatment was compared to fenbendazole treatment [16]. Other molecules have shown efficacy in treating canine giardiasis (ronidazole, oxfenbendazole, nitazoxanide) [7, 17–20]. A potential limitation in the study design could be the absence of a group receiving treatment without a concurrent hygiene protocol. However, the application of hygiene measures across both groups supports the argument that the observed significant difference in efficacy is primarily attributable to AYRADIA. Furthermore, this approach reflects standard veterinary practice, where hygiene measures are considered an integral component of giardiasis management [7, 10, 11].

The observation of *G. duodenalis* positivity in dogs without overt clinical signs (diarrhea) underscores the importance of routine screening, particularly in high-risk populations such as puppies and multi-dog households [14, 21]. Rapid tests offer convenient screening in veterinary clinics (e.g., IDEXX SNAP Giardia Test, Virbac Speed Giardia test) [13, 22]. However, positive results require careful interpretation, as they do not confirm active infection or assess treatment success [7]. Understanding each test's performance metrics (sensitivity, specificity, positive and negative predictive values) is crucial for interpreting results accurately within specific clinical contexts [13, 21]. Direct fluorescent antibody (DFA) testing remains the recommended diagnostic gold standard [14].

The availability of metronidazole formulations specifically designed for canine use is limited, and no other liquid formulations currently exist. The availability of AYRADIA as a liquid oral suspension provides a significant advantage in veterinary practice. This liquid oral suspension facilitates precise, weight-based dosing, particularly crucial for small-breed dogs where tablet splitting can lead to inaccurate administration. Liquid oral suspensions facilitate precise dosing, minimizing the risks associated with inaccurate dosing, such as potential side effects from overdosing or the development of pathogen resistance due to underdosing [14, 19].

Effective management of canine giardiasis is critical for safeguarding both animal and human health. The zoonotic potential of *G. duodenalis* necessitates prompt and effective treatment to minimize the risk of transmission to human populations [4, 5, 14]. Furthermore, the economic burden associated with prolonged giardiasis, including veterinary costs and potential impact on animal performance, underscores the importance of effective therapeutic interventions [2, 7, 11, 14].

## Conclusions

Based on the results of this study, AYRADIA dosed at 0.2 ml/kg twice daily for 5 days is effective for treating *G. duodenalis*, with a positive safety profile. Given the challenges associated with treating *G. duodenalis*, particularly in terms of relapse, the flavored oral suspension eases the administration by accurate weight-based dosing and high acceptance. When used according to the product label, AYRADIA/ERADIA is a valuable option for the therapeutic management of giardiasis in veterinary practice.

## Data Availability

No datasets were generated or analyzed during the current study.
